# Spatio-temporal evolution and driving factors of the coupling and coordinated development of China's digital economy and older adult care services

**DOI:** 10.3389/fpubh.2025.1490461

**Published:** 2025-02-26

**Authors:** Hui Liu, Wei Wang, Sha Li

**Affiliations:** College of Public Administration and Law, Hunan Agricultural University, Changsha, China

**Keywords:** digital economy, older adult care services, entropy method, coupling coordination degree model, geodetector model

## Abstract

**Background:**

This study aims to examine the level of coupled and coordinated development between China's digital economy and older adult care services, analyzing their spatiotemporal evolution characteristics and key influencing factors, with the goal of providing feasible recommendations and scientific bases for the development of the digital economy and older adult care services in China.

**Methods:**

This study uses publicly available panel data from China for the years 2015–2022. It employs the entropy method to measure the weights of various indicators in the digital economy and older adult care services. The study analyzes the level of coordinated development between the two using the coupling coordination degree model, and measures the main driving factors using the geographical detector model.

**Results:**

(1) The overall level of coupling and coordinated development between China's digital economy and older adult care services shows an upward trend, but the growth rate is uneven, exhibiting an “M-shaped” pattern, with rapid growth followed by gradual slowdown, a bottoming-out rebound, and then a continuous decline. (2) There are significant spatial differences in the coupling and coordinated development of China's digital economy and older adult care services. Coastal areas are developing rapidly, inland areas have great potential, while peripheral areas are relatively lagging behind. Additionally, neighboring regions show regional linkage dynamics. (3) The main factors driving the coupling and coordinated development of China's digital economy and older adult care services include enterprise website ownership, technological contract turnover, the proportion of information technology service income, the building area of older adult care institutions, daily in-house visits, and the number of professional technical personnel.

**Conclusion:**

To achieve coordinated development between the digital economy and older adult care services, efforts should focus on policy, market, technology, and talent. The government should support technological innovation and new service models, while tailoring strategies to regional market demands. Additionally, accelerating the industrialization of innovations and promoting intelligent upgrades in older adult care services are crucial. Finally, more investment is needed to cultivate composite talents in both the government and older adult care institutions.

## 1 Introduction

According to the “2022 Civil Affairs Development Statistical Bulletin” released by China's Ministry of Civil Affairs, by the end of 2022, the older adult population aged 60 and above in China had reached 280 million, accounting for 19.8% of the total population, with an annual growth rate of 4.7%. This data not only highlights the vast scale of China's older adult population but also signals the accelerating process of population aging. Population aging is quietly reshaping the social structure, leading to a sharp increase in demand for older adult care services (1). However, with the decline in physical health and the increasing vulnerability of older adults in terms of mental wellbeing, the demand for older adult care services is no longer limited to medical care but also increasingly includes daily care and emotional support (2).

Specifically, in terms of medical services, due to the decline in physical functions, older adults have a more frequent and urgent need for medical services (3). However, current medical resources are relatively limited and unevenly distributed, leading to issues such as difficulty in seeing a doctor and high medical costs (4). Older adults often have to wait a long time to see a doctor, and the high cost of healthcare creates a heavy economic burden for both the older adults and their families (5). In terms of daily care, as older adults experience a gradual decline in physical functions, their need for daily care has been growing (6). However, the current older adult care service system faces prominent issues, such as insufficient professional skills of care staff and a lack of detailed service attitudes (7).

These problems directly hinder the effective fulfillment of older adults' diverse care needs, which in turn affects their quality of life (8). In terms of emotional support, many older adults feel increasingly lonely and anxious due to factors like their children being busy with work and shrinking social circles (9). However, current older adult care providers often focus on medical services and neglect their emotional needs. This neglect leads to a lack of necessary comfort and support for older adults, further impacting their physical and mental health (10). Fortunately, the rise of the digital economy offers new solutions to address these challenges. Specifically, the digital economy can provide more convenient access to medical services for older adults through online consultations and telemedicine, reducing the burden of seeking medical care (11).

At the same time, the digital economy can use machine algorithms and artificial intelligence to learn older adults' behavior preferences and automatically adjust daily care plans according to their needs (12). For example, smart care devices can automatically adjust the hardness of the bed and the height of the pillow based on older adults' activity levels and sleep quality, offering a more comfortable sleeping environment (13). Additionally, the digital economy can provide emotional support for older adults through smart companion robots and online social platforms (14).

These technologies can simulate human emotional communication, interacting with older adults to alleviate their feelings of loneliness and anxiety (15). In view of this, in 2022, the State Council issued the “14th Five-Year Plan for Digital Economy Development,” which explicitly stated: “We should make full use of the powerful driving force of the digital economy to actively address the challenges brought about by population aging, and promote the digital transformation and upgrading of the older adult care service system.” This strategic guideline elaborates on the application prospects of the digital economy in the field of older adult care services, elevating the importance of integrating the digital economy with older adult care services to a new level.

## 2 Literature review

Scholars generally believe that older adult care services, as an effective response measure, have core value in meeting the diverse living needs of the older adults. However, with the rapid development of the social economy, the demand for older adult care services has gradually shifted from traditional areas such as medical care and health services to more diversified fields like daily care and emotional support (16). This change not only reflects older adults' increasing pursuit of a better quality of life but also poses higher demands on the older adult care service system. To build a more comprehensive older adult care service system, China has established a multi-level system that includes family, community, institutions, and government (17). These components not only differ significantly in function but also play different roles as the economy and society develop. Among them, family care is the most fundamental and efficient model.

However, with the acceleration of urbanization, more and more rural children choose to work in cities, leaving many older adults in rural areas to live alone (18). Research shows that older adults living in solitude in rural areas face increasingly serious declines in their quality of life, and even show tendencies toward suicide (19). The weakening of the traditional family care model has led older adults individuals to make more choices, seeking help from other parties, which led to the emergence of community-based older adult care (20). Community older adult care is a model that provides services such as daily care, housekeeping, rehabilitation, emotional support, and cultural activities to older adults within a community (21).

Compared to traditional family care, community-based older adult care allows older adults to live in a familiar community environment, enjoying the warmth and care of a family while receiving specialized and diverse services provided by the community (22). However, community older adult care is still in its early stages due to unstable funding sources and inadequate infrastructure (23). Institutional older adult care is a model where third-party social organizations provide services. Compared to community care, it has a more stable funding source and can hire professional caregivers to provide services (24).

However, due to high costs and the attachment to one's home, only a small number of older adults opt for institutional care such as nursing homes (25). As for government-based older adult care, it is an important component of the public older adult care service system. It mainly provides basic living security and older adult care services through policy formulation, financial support, and the construction of public facilities (26). The government care model plays a critical role in meeting the basic living needs of older adults, especially for those facing economic difficulties or those without children or with children who are unable to care for them (27). It serves as an important pillar for their support.

The digital economy is widely recognized as the major economic form following the agricultural and industrial economies. Its core lies in the use of digitalized information as a key production factor, relying on modern information networks as transmission media, and efficiently utilizing digital technologies to achieve precise resource allocation, thereby driving the economy toward higher-quality development (28). Compared to traditional economic models, the digital economy demonstrates unprecedented potential, not only promoting the optimization and upgrading of industrial structures but also fostering numerous emerging business models through cross-sectoral innovative strategies, significantly enhancing the potential for economic growth (29).

In particular, the empowerment of the digital economy in the field of older adult care services is especially notable. On one hand, the digital economy has created comprehensive older adult care service platforms that integrate resources from multiple sectors, such as older adult care institutions, medical facilities, rehabilitation centers, and tourism centers, digitally (30). This has formed a multi-functional, all-encompassing older adult care service ecosystem that covers health management, daily care, leisure, entertainment, and more (31). This optimization and upgrading of industrial structures not only significantly improves the efficiency of older adult care services but also greatly enriches the content of these services, making them more aligned with the diverse needs of older adults, thereby enhancing their quality of life and wellbeing.

On the other hand, the digital economy has promoted the cross-sectoral integration of older adult care services with fields such as healthcare, tourism, and finance, giving rise to new service models like “medical and older adult care integration,” developing “older adult tourism” products, and innovating “long-term care insurance” financial services (32). These achievements not only significantly improve the professionalism of older adult care services but also drive innovation in the older adult care industry, laying a solid foundation for building a more comprehensive older adult care system.

Summarizing previous research, studies on older adult care services initially focused more on the basic content and development models of older adult care services, with little emphasis on the digital economy. However, with the rapid development of digital technologies, the digital economy has gradually become an important economic form. As a result, studies directly addressing the relationship between older adult care services and the digital economy have increased, but most of them are still confined to theoretical research on the integration of digital economy and older adult care services, lacking empirical analysis of real-world situations. This significantly limits our comprehensive understanding of the integration and development of the digital economy and older adult care services, thereby hindering the formulation and implementation of related policies and ultimately affecting the pace and effectiveness of their integrated development.

Therefore, based on a systematic review of existing literature, this paper selects 31 provinces, autonomous regions, and municipalities in China from 2015 to 2022 as the research object and constructs a comprehensive evaluation index system for the digital economy and older adult care services. The entropy method is used to assign weights to the indicators in the index system and to measure the comprehensive scores of the digital economy and older adult care services. Then, the coupling coordination degree model is employed to analyze the coupling coordination degree value between the digital economy and older adult care services and examine its evolutionary characteristics in terms of time and space. Finally, the geographical detector model is used to analyze the main influencing factors. This study aims to provide theoretical references and policy recommendations for the coordinated development of the digital economy and older adult care services in different regions.

## 3 Materials and methods

### 3.1 Data source

This study uses publicly available panel data from China for the years 2015–2022, covering 31 provinces, autonomous regions, and municipalities. Due to the unavailability of open data from Hong Kong, Macau, and Taiwan, these regions have been excluded from the study to ensure research rigor and data accuracy. The raw data primarily comes from authoritative sources such as the National Bureau of Statistics, provincial (municipal, autonomous region) statistical bureaus, and statistical yearbooks. It is worth noting that although there were some missing data during the processing, linear interpolation methods were used to reasonably fill in these gaps, and this treatment does not affect the accuracy and reliability of the analysis results.

### 3.2 Model method

#### 3.2.1 Entropy method

The entropy method is an objective weighting method based on information entropy theory. The principle is to measure the amount of information contained in an indicator by calculating its information entropy, and then determine the weight of each indicator in the comprehensive evaluation (33). Smaller information entropy indicates a greater degree of variation in the indicator and more information provided, resulting in a higher weight in the comprehensive evaluation. Conversely, when the information entropy is large, it indicates a smaller degree of variation in the indicator and less information contained, leading to a corresponding decrease in weight in the comprehensive evaluation. In this study, the entropy method is used to determine the weights of various indicators in the digital economy and older adult care services. Compared to other methods, the advantage of the entropy method lies in avoiding the arbitrariness and inaccuracy of subjective weighting, and it can automatically adjust weights based on the characteristics of the data, making the evaluation results closer to the actual situation. The specific steps are as follows:

(1) Data standardization processing:


(1) $$\begin{array}{l} S=\frac{X-Mean}{Std} \end{array}$$

Where, S is the standardized data; X is the original data; Mean is the mean of the original data; Std is the standard deviation of the original data.

(2) Calculating indicator weights:


(2) $$P_{ij}=\frac{X_{ij}^{'}}{\sum_{i=1}^{n}X_{ij}^{'}}$$

Where, i is the i-th year; j is the j-th indicator; n is the number of years.

(3) Calculating indicator entropy:


(3) $$\begin{array}{l} E_{j}=-k\overset{n}{\underset{i=1}{\sum }}P_{ij}\ln P_{ij} \end{array}$$

Where, k=1/ln^*n*^; when *P*_*ij*_=0, let *P*_*ij*_ln_*ij*_=0

(4) Calculating information efficiency value:


(4) $$\begin{array}{l} D_{j}=1-E_{j} \end{array}$$

Where, 0 ≤ *E*_*j*_ ≤ 1.

(5) Calculating weight results:


(5) $$\begin{array}{l} W_{j}=1-\frac{E_{j}}{\sum_{j=1}^{m}D_{j}} \end{array}$$

#### 3.2.2 Coupling coordination model

The coupling coordination degree model is a mathematical model used to assess the interaction and coordinated development degree between two or more systems. The basic principle of this model is based on the dynamic behavior and interaction relationships between systems, revealing the correlation and coordinated development status through quantitative analysis (34). This model is widely applied in fields such as economics, society, and the environment for coordinating development evaluation. For example, in regional economic research, it can be used to evaluate the interaction and coordinated development level between different economic sectors; in ecological and environmental protection, it can be used to assess the coordination between economic development and environmental protection. The model involves the calculation of three indicators: coupling degree (C value), coordination index (T value), and coupling coordination degree (D value). The coupling degree (C value) refers to the level of interaction between two or more entities, reflecting the degree of mutual dependence and constraints. The coordination index (T value) reflects the degree of positive coupling within the coupled system, that is, whether the systems develop harmoniously and coordinately together. It represents the quality of the coordination between the systems. The coupling coordination degree (D value) is a comprehensive reflection of the coupling degree (C value) and the coordination index (T value), used to comprehensively evaluate the coordinated development degree between systems. In this paper, the coupling coordination degree model is used to calculate the coordinated development level between the digital economy and older adult care services. Compared to other methods, the coupling coordination degree model not only reflects the degree of interaction between systems but also reveals the coordination relationship between the internal elements of the systems, making it a very effective tool for analyzing complex issues. The specific formula is as follows:


(6) $$\begin{array}{l} {C=\left\{\frac{U_{1}\times U_{2}}{{\left(U_{1}+U_{2}\right)}^{2}}\right\}}^{\frac{1}{2}} \end{array}$$

(7) $$\begin{array}{l} D=\sqrt{C\times T}\;,T=\sqrt{\alpha U_{1}\times \beta U_{2}}\;,\alpha +\beta =1 \end{array}$$

Where, C represents coupling degree, T represents the coordination index, D represents coordination degree, *U*_1_ and *U*_2_ represent the scores of the comprehensive indices for the digital economy and older adult care services respectively; α and β are undetermined weights, because both are equally important, so α = β = 0.5. According to the customary division standards for coordination research, coordination can be divided into ten categories for discussion, as shown in Table 1.

**Table 1:** Criteria for classifying the degree of coupling coordination.

**Coordination degree D**	**Value coordination type**	**Coordination degree D**	**Value coordination type**
0.0–0.1	Extreme discrepancy	0.5–0.6	Slight coordination
0.1–0.2	Severe discrepancy	0.6–0.7	Mild coordination
0.2–0.3	Moderate discrepancy	0.7–0.8	Moderate coordination
0.3–0.4	Mild discrepancy	0.8–0.9	High coordination
0.4–0.5	Slight discrepancy	0.9–1.0	Very High coordination

#### 3.2.3 Geodetector model

The geographic detector model is a statistical method used to explore spatial differentiation and the underlying influencing factors. Its core idea is based on the following assumption: if an independent variable has a significant impact on a dependent variable, then the spatial distribution of the independent and dependent variables should exhibit similarity. The geographic detector quantifies the explanatory power of independent variables on the spatial distribution of the dependent variable, revealing the underlying influencing factors of geographic phenomena (35). This model is widely applicable and can be effectively used in fields such as land use, regional economy, and environmental protection. For example, in the field of land use, the geographic detector model can help identify which factors influence the way land is used. In the regional economy field, it can be used to identify the key factors promoting or restricting regional economic development.

The specific calculation of the geographic detector model includes four components: factor detection, interaction detection, ecological detection, and risk zone detection. Factor detection assesses the explanatory power (q value) of each factor on the spatial differentiation of the dependent variable, evaluating the role of each factor in the spatial distribution of geographic phenomena. The higher the q value, the stronger the explanatory power of the factor on the dependent variable. Interaction detection compares the q value when two factors interact with the q values when they act individually, to assess the influence of their interaction on the spatial distribution of the geographic phenomenon. If the interaction increases the explanatory power, it indicates a synergistic effect between the two factors; if it decreases the explanatory power, it suggests an antagonistic effect.

Ecological detection is used to compare whether there are significant differences in the influence of different factors on the spatial distribution of the dependent variable, providing strong evidence for the relative importance of factors. Risk zone detection focuses on determining whether there are significant differences in the attribute means between different sub-regions, offering a more detailed description of the spatial distribution characteristics of geographic phenomena. In this study, we primarily applied the factor detection component of the geographic detector model to analyze the main influencing factors of the coupled and coordinated development of the digital economy and older adult care services. Compared with other methods, the geographic detector model performs exceptionally well in analyzing small sample data, providing more accurate and reliable research results. The specific formulas are as follows:


(8) $$\begin{array}{l} q=1-\frac{1}{N\sigma^{2}}\overset{L}{\underset{h=1}{\sum }}N_{h}\sigma_{h}^{2} \end{array}$$

Where q is the detection value of the factor detector; h=1; …L represents the stratification of variable X; N and *N*_*h*_ are the sample sizes of the study area and the detection area, respectively; σ^2^ and $\sigma_{h}^{2}$ are the variances of Y in the study area and detection area, respectively. The value of q ranges from 0 to 1, and a higher q value indicates a stronger explanatory effect of factor X on Y.

### 3.3 Variables

To further explore the coupled and coordinated development of China's digital economy and older adult care services, this paper constructs a comprehensive evaluation index system that includes two subsystems: digital economy and older adult care services. These two subsystems are independent yet interrelated, collectively forming a complete framework for evaluating the coupled and coordinated development of digital economy and older adult care services.

The digital economy subsystem can be further divided into three levels: information communication, technological innovation, and industrial applications. This is mainly because information communication, as a key pillar of the digital economy, undertakes core responsibilities such as data transmission, storage, and processing (36). It serves as the “highway” for data flow and directly affects the operational efficiency and development potential of the digital economy. Without efficient, stable, and secure information communication networks, the digital economy cannot achieve rapid and accurate data transmission and sharing, and its advantages would be unattainable. Therefore, when constructing the comprehensive evaluation index system for the digital economy, this study places particular emphasis on the consideration of information communication to assess the hardware infrastructure construction and its level of completeness in different regions. This level includes a series of core indicators, such as the number of broadband internet users (37), mobile phone penetration rate (38), the length of long-distance optical fibers per unit area (39), and the ownership rate of corporate websites (40). Through these indicators, we can comprehensively measure the extent of information communication coverage, transmission speed, and the level of enterprise informatization in different regions, providing data support for subsequent research.

Technological innovation is the core driving force of the digital economy and a key factor in promoting its sustained, rapid, and healthy development (41). It not only fosters new industrial models but also drives the transformation and upgrading of traditional industries, enhancing industrial value-added and competitiveness. Therefore, when constructing the comprehensive evaluation index system for the digital economy, this study considers technological innovation as an important dimension, aiming to assess the strength and potential of different countries or regions in the field of technological innovation. This level includes a series of core indicators, such as R&D expenditures of large industrial enterprises (40), the total value of technology contract transactions (39), and the number of patent applications and grants. Through these indicators, we can comprehensively measure the investment intensity in technological innovation, the efficiency of technological achievement conversion, and the strength of intellectual property protection in different regions, providing data support for subsequent research.

Industrial applications are the key link to realizing the value of the digital economy and an important manifestation of the deep integration of the digital economy with the real economy (42). The widespread penetration of the digital economy in industrial applications has not only brought great convenience to people's production and daily life but also significantly enhanced the level of economic development. Therefore, when constructing the comprehensive evaluation index system for the digital economy, this study considers industrial applications as an independent and important dimension, aiming to comprehensively demonstrate the practical outcomes of the digital economy in industrial applications. This level includes a series of core indicators, such as the proportion of information technology services revenue to GDP (42), the proportion of e-commerce revenue from enterprises to GDP (36), and the proportion of telecommunications revenue to GDP (43). Through these indicators, we can intuitively reflect the actual value generated by the digital economy in the present, providing data support for subsequent research.

The older adult care service subsystem can also be divided into three levels: infrastructure, service capacity, and organizational management. This is mainly because infrastructure, as the material foundation of older adult care services, is directly related to the quality of service delivery (44). Well-developed infrastructure can provide older adults with a comfortable and safe living environment, serving as the foundation for improving the level of older adult care services. Therefore, when constructing the comprehensive evaluation index system for older adult care services, this study considers infrastructure as a key dimension, aiming to comprehensively assess the construction status of infrastructure in older adult care services. This includes a series of core indicators, such as the number of older adult care institutions (45), the number of beds at the end of the year (46), and the floor area of the institutions (44). These indicators can intuitively reflect the effectiveness of infrastructure construction in older adult care institutions, providing reliable data support for the research.

Service capacity is the core component of older adult care services, directly affecting the quality of life care, health protection level, and psychological satisfaction of older adults (47). Good service capacity helps improve the happiness of older adult individuals, enhances the competitiveness of older adult care institutions, and promotes the healthy development of the older adult care service industry. Therefore, when constructing the comprehensive evaluation index system for older adult care services, this study views service capacity as an indispensable dimension, aiming to comprehensively assess the actual situation of service capacity in older adult care services. A series of core indicators includes the number of people in the facility per day (48) and the number of people attending rehabilitation and medical outpatient services (49). These indicators can accurately reflect the actual service capacity of older adult care institutions, providing data support for in-depth research.

Organizational management is an important guarantee for older adult care services, directly affecting the stability and sustainability of these services (50). Good organizational management ensures the standardized and efficient operation of older adult care services, providing older adult individuals with long-term, stable, and high-quality care. Therefore, when constructing the comprehensive evaluation index system for older adult care services, this study places organizational management in a prominent position, aiming to comprehensively assess the level of organizational management in older adult care institutions. Core indicators include the number of management staff (51), the number of professional and technical personnel (52), and the number of social workers (53). These indicators can intuitively reflect the management hierarchy, technical support, and community service potential of older adult care institutions, thereby providing data support for in-depth research. The specific results are shown in Table 2.

**Table 2:** Evaluation indicator system for coupled and coordinated development of digital economy and older adult care services in China.

**Primary indicator**	**Secondary indicator**	**Tertiary indicator**	**Unit**
Digital economy	Information and communication	Internet broadband access users	Ten thousand households
		Mobile phone penetration rate	Units per hundred people
		Length of long-distance optical fiber per unit area	Ten thousand kilometers
		Number of enterprise websites per hundred enterprises	Units
	Technological innovation	R&D expenditure of large-scale industrial enterprises	Ten thousand yuan
		Total amount of technology contract transactions	Ten thousand yuan
		Number of patents applied and granted	Items
	Industrial application	Proportion of information technology service revenue to GDP	Percent
		Proportion of enterprise e-commerce to GDP	Percent
		Proportion of telecommunications service volume to GDP	Percent
Older adult care services	Infrastructure	Number of older adult care institutions	Units
		End-of-year bed capacity	Sheets
		Institutional building area	Square meters
	Service capacity	Daily number of residents in the facility	Times
		Rehabilitation and medical outpatient visits	Times
	Organizational management	Number of managers	People
		Number of professional technical staff	People
		Number of social workers	People

## 4 Results

After conducting a thorough analysis, the findings regarding the coupling coordination degree between the digital economy and older adult care services in China are presented in Table 3. This table offers a valuable insight into the index of China's coupled and coordinated development level within these two vital domains.

**Table 3:** Index of China's coupled and coordinated development level of the digital economy and older adult care services.

**Region**	**2015**	**2016**	**2017**	**2018**	**2019**	**2020**	**2021**	**2022**
Beijing	0.103	0.151	0.573	0.627	0.675	0.825	0.775	0.995
Tianjin	0.226	0.257	0.206	0.222	0.841	0.988	0.908	0.92
Hebei	0.1	0.258	0.452	0.474	0.745	0.928	0.955	0.993
Shanxi	0.317	0.21	0.392	0.238	0.555	0.706	0.82	0.995
Inner Mongolia	0.226	0.3	0.568	0.658	0.723	0.563	0.697	0.3
Liaoning	0.198	0.292	0.212	0.487	0.734	0.84	0.945	0.995
Jilin	0.137	0.29	0.66	0.872	0.93	0.956	0.855	0.935
Heilongjiang	0.307	0.147	0.399	0.251	0.661	0.85	0.947	0.976
Shanghai	0.1	0.341	0.433	0.597	0.72	0.836	0.895	0.995
Jiangsu	0.231	0.341	0.444	0.241	0.742	0.917	0.93	0.92
Zhejiang	0.1	0.271	0.452	0.64	0.777	0.891	0.931	0.995
Anhui	0.1	0.256	0.407	0.568	0.667	0.854	0.907	0.992
Fujian	0.1	0.174	0.378	0.407	0.641	0.77	0.922	0.995
Jiangxi	0.1	0.242	0.296	0.401	0.62	0.807	0.873	0.995
Shandong	0.291	0.496	0.337	0.226	0.65	0.83	0.851	0.995
Henan	0.1	0.216	0.24	0.384	0.669	0.855	0.898	0.995
Hubei	0.23	0.444	0.505	0.247	0.705	0.886	0.933	0.982
Hunan	0.208	0.272	0.369	0.221	0.663	0.858	0.908	0.969
Guangdong	0.1	0.302	0.467	0.53	0.761	0.916	0.955	0.982
Guangxi	0.1	0.228	0.387	0.515	0.619	0.783	0.904	0.962
Hainan	0.501	0.244	0.333	0.753	0.283	0.813	0.931	0.954
Chongqing	0.1	0.397	0.321	0.468	0.599	0.691	0.828	0.995
Sichuan	0.267	0.4	0.578	0.252	0.697	0.831	0.877	0.995
Guizhou	0.315	0.339	0.209	0.414	0.586	0.666	0.664	0.636
Yunnan	0.235	0.19	0.457	0.474	0.736	0.805	0.872	0.995
Tibet	0.315	0.271	0.332	0.243	0.488	0.708	0.865	0.995
Shaanxi	0.1	0.335	0.412	0.438	0.794	0.911	0.967	0.995
Gansu	0.271	0.337	0.582	0.237	0.505	0.705	0.932	0.945
Qinghai	0.431	0.305	0.187	0.256	0.76	0.914	0.91	0.831
Ningxia	0.103	0.167	0.367	0.384	0.608	0.746	0.897	0.995
Xinjiang	0.127	0.324	0.316	0.22	0.459	0.644	0.83	0.995

### 4.1 Temporal characteristic analysis of the coordinated development of China's digital economy and older adult care services

The coupling coordination development between China's digital economy and older adult care services has shown an overall upward trend, but with uneven growth rates, displaying an “M-shaped” pattern of “rapid growth—gradual slowdown—bottoming out and recovery—sustained decline.” Specifically, during the rapid growth phase (2015–2016), the coupling coordination degree between the digital economy and older adult care services rose sharply from 0.198 to 0.284, with a growth rate of 43.43%, reaching a moderately imbalanced level. This indicates that in this phase, the initial integration of the digital economy and older adult care services yielded significant results, with both mutually promoting each other, entering a prosperous development period. In the gradual slowdown phase (2016–2018), although the coupling coordination degree continued to rise from 0.284 to 0.418, the growth rate gradually slowed, with rates of 39.44% and 5.56%, entering a mildly imbalanced level. Among them, the increase from 0.396 to 0.418 was the smallest growth during the entire development phase.

This suggests that during this phase, the integration of the digital economy and older adult care services faced challenges, and the growth rate slowed significantly, entering a bottleneck period. In the bottoming out and recovery phase (2018–2019), the coupling coordination degree surged from 0.418 to 0.665, with a growth rate of 60.05%, jumping to a lightly coordinated level, marking the largest increase in the entire development process. This indicates that in this phase, the integration of the digital economy and older adult care services made breakthrough progress, with a significant enhancement in their synergistic effects, entering the golden period of development.

**Figure 1:**
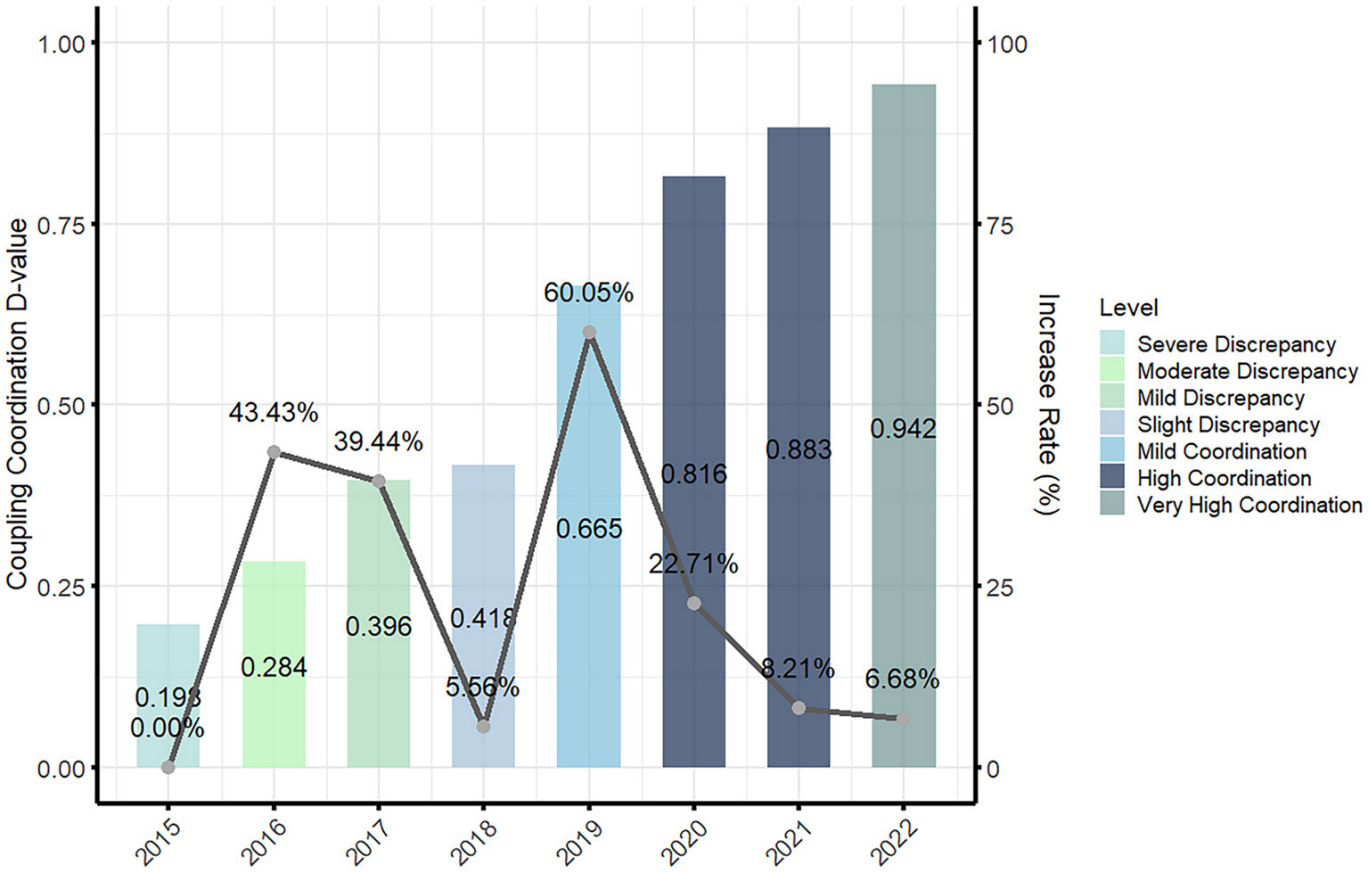
Temporal distribution of the coupled and coordinated development of digital economy and older adult care services in China.

In the sustained decline phase (2019–2022), although the coupling coordination degree continued to rise from 0.665 to 0.942, reaching an extremely coordinated level, the growth rate gradually decreased, with rates of 22.71%, 8.21%, and 6.68%. This indicates that during this phase, the integration of the digital economy and older adult care services was influenced by other factors, and the growth rate continued to decline, entering a period of developmental decline. The specific situation is shown in Figure 1.

### 4.2 Spatial characteristic analysis of the coordinated development of China's digital economy and older adult care services

The spatial disparity in the coupling and coordinated development of China's digital economy and older adult care services is significant, forming a diverse spatial development pattern: rapid development in coastal areas, huge potential in inland areas, and relative stagnation in peripheral regions. In this process, the geographical proximity effect is notable, with neighboring regions depending on and promoting each other's development, creating a regional linkage development trend. Specifically, Zhejiang, Guangdong, and Shanghai, as typical representatives of coastal areas, have coupling coordination values of 0.6321, 0.6266, and 0.6146, respectively, which are at a mild coordination level, fully demonstrating these regions' leadership in the integration of the digital economy and older adult care services, making them role models nationwide. Central regions such as Shaanxi, Hubei, and Sichuan also perform well, with coupling coordination values of 0.6190, 0.6165, and 0.6121, respectively, also at a mild coordination level.

**Figure 2:**
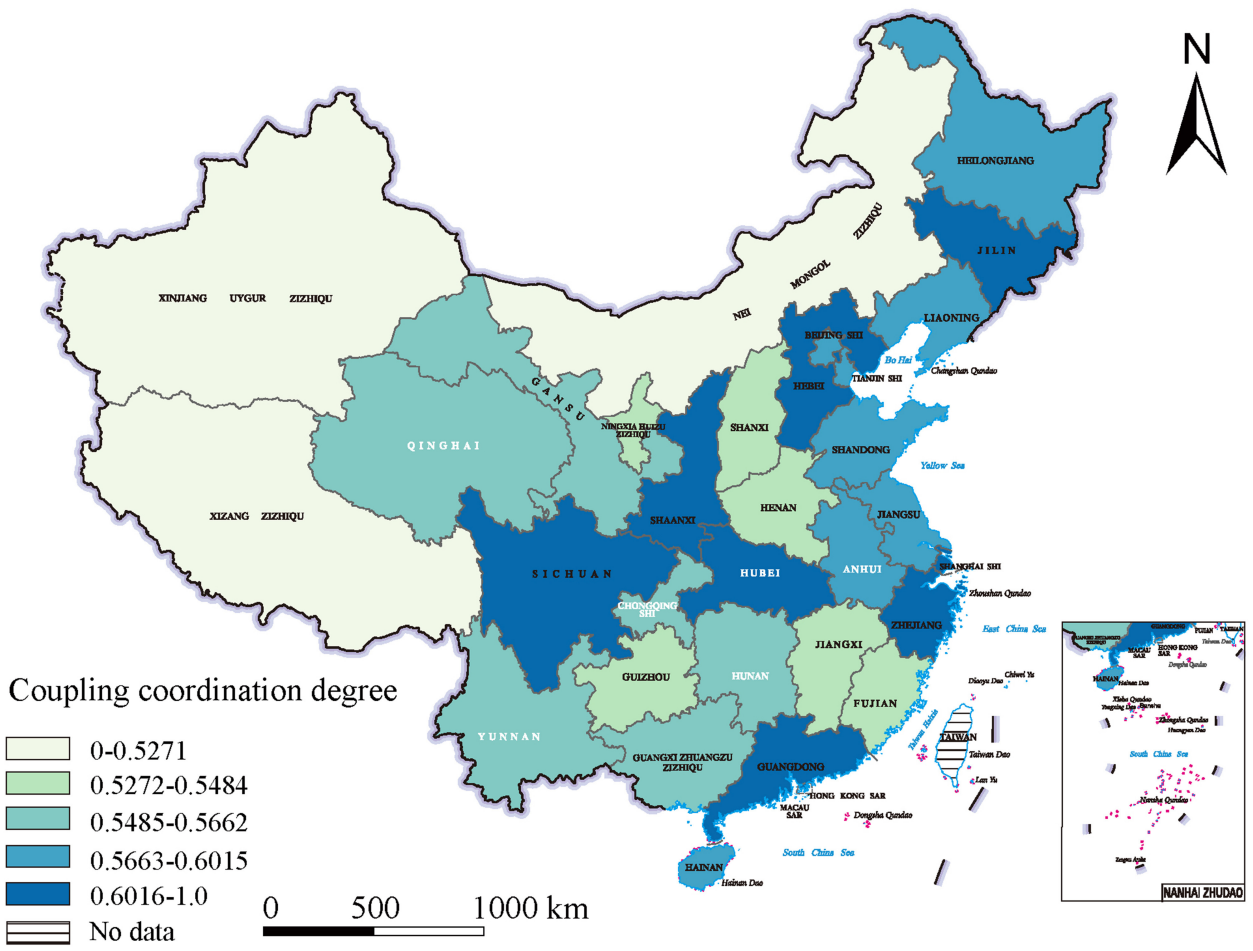
Spatial distribution of the coupled and coordinated development of digital economy and older adult care services in China.

These regions, leveraging their multiple advantages, have shown strong development momentum in the coordinated development of the digital economy and older adult care services, becoming some of the most promising regions for future development. However, peripheral regions like Tibet, Inner Mongolia, and Xinjiang have relatively low coupling coordination values of 0.5271, 0.5044, and 0.4894, respectively, at a weak coordination or even weak disorder level. These regions face multiple real-world constraints, causing a slower integration of the digital economy and older adult care services. They need to gradually narrow the gap with more developed regions and improve their integration development level in the future. Notably, the geographical proximity effect in the coupling and coordinated development of the digital economy and older adult care services is fully reflected between provinces. Taking Shanghai as an example, with a coupling coordination value of 0.6146, Shanghai not only achieves its own prosperity but also generates a significant radiating effect on neighboring regions such as Jiangsu and Anhui, driving their levels of digital economy and older adult care services integration to 0.5958 and 0.5939, respectively. This regional linkage development model provides valuable lessons and inspiration for regions across the country. The specific results are shown in Figure 2.

### 4.3 Analysis of driving factors in the coordinated development of China's digital economy and older adult care services

This study hypothesizes that information communication, technological innovation, industrial applications, infrastructure, service capabilities, and organizational management all influence the coupling and coordinated development level of the digital economy and older adult care services. The dependent variable in this study is the coupling coordination degree between the digital economy and older adult care services, while the independent variables include information communication, technological innovation, industrial applications, infrastructure, service capabilities, and organizational management. Specifically, information communication is measured by indicators such as the number of internet broadband access users (*X*_1_), mobile phone penetration rate (*X*_2_), the length of long-distance optical cables per unit area (*X*_3_), and the number of websites per 100 enterprises (*X*_4_), with the highest q-value for the number of websites per 100 enterprises (*X*_4_) being 0.3542.

Technological innovation is evaluated by the R&D expenditure of industrial enterprises above a designated size (*X*_5_), the total value of technology contract transactions (*X*_6_), and the number of patents granted (*X*_7_), with the highest q-value for total technology contract transaction value (*X*_6_) being 0.3025. Industrial applications are measured by the proportion of information technology service revenue in GDP (*X*_8_), the proportion of e-commerce in GDP (*X*_9_), and the proportion of telecommunications business volume in GDP (*X*_10_), with the highest q-value for the proportion of information technology service revenue in GDP (*X*_8_) being 0.5072.

**Figure 3:**
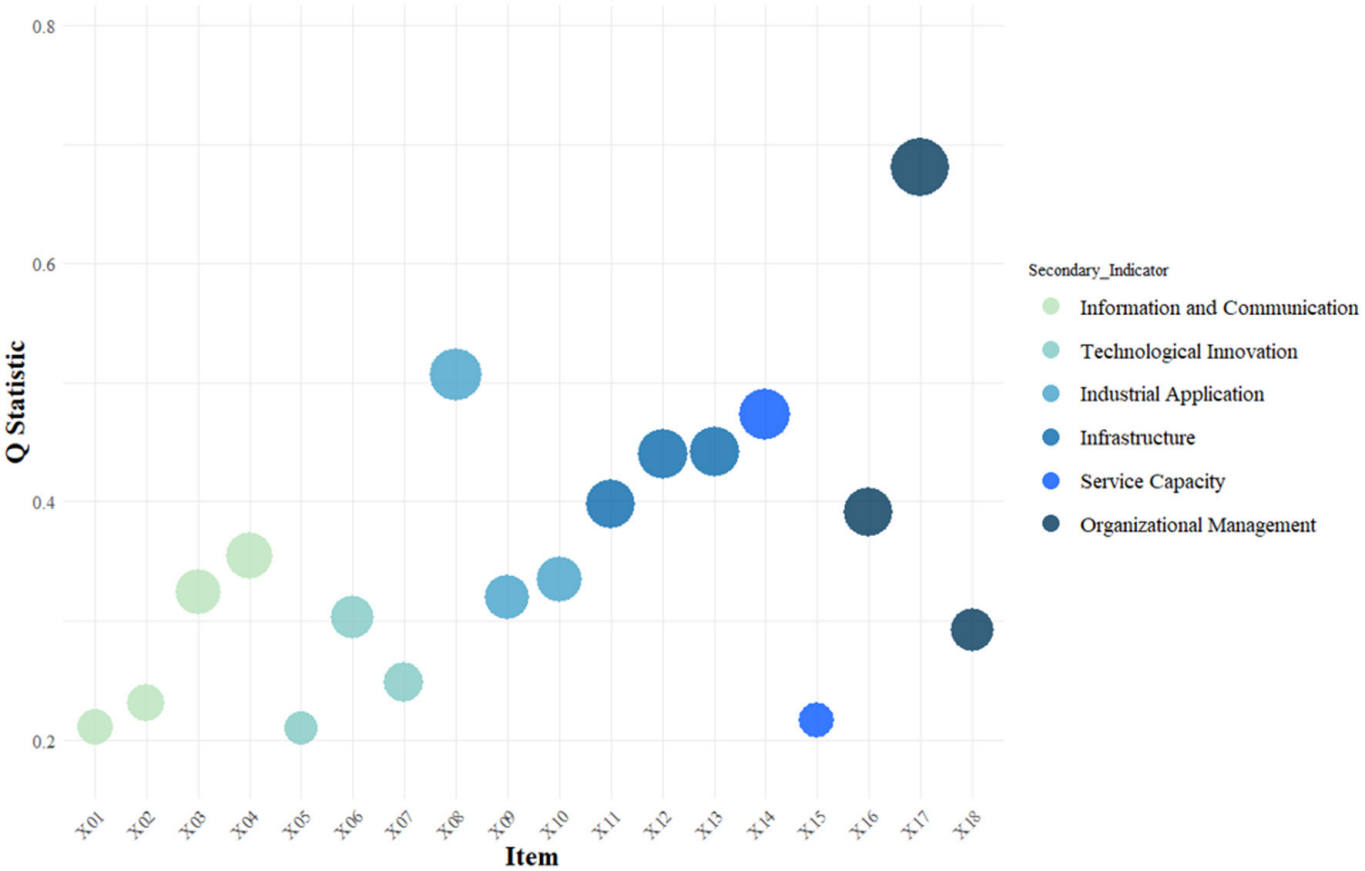
Analysis of driving factors for the coupled and coordinated development of digital economy and older adult care services in China.

Infrastructure is assessed by the number of older adult care institutions (*X*_11_), the number of beds at the end of the year (*X*_12_), and the building area of institutions (*X*_13_), with the highest q-value for building area (*X*_13_) being 0.4422. Service capabilities are represented by the number of people in care institutions per day (*X*_14_) and the number of rehabilitation and medical outpatient visits (*X*_15_), with the highest q-value for the number of people in care institutions per day (*X*_14_) being 0.4733. Organizational management is measured by the number of management personnel (*X*_16_), the number of professional technical personnel (*X*_17_), and the number of social workers (*X*_18_), with the highest q-value for the number of professional technical personnel (*X*_17_) being 0.6806. The specific results are shown in Figure 3.

## 5 Discussion

### 5.1 Key findings

The coupling and coordinated development level of China's digital economy and older adult care services has shown an overall upward trend, but the growth rate is uneven, presenting an “M-shaped” fluctuation pattern with characteristics of “rapid growth—gradual slowdown—bottoming out and recovery—continuous decline.” The reasons for this phenomenon can be summarized as follows:

(1)Policy guidance is the core driving force in the rapid growth stage. In July 2015, the State Council issued the “Guiding Opinions on Actively Promoting the 'Internet +' Action,” which clearly outlined the important direction for promoting the development of the smart older adult care industry. The policy advocated fully utilizing existing internet resources to build a comprehensive older adult care information service network, aiming to provide diversified, all-encompassing home care services for older adults, including nursing, health management, rehabilitation care, and more. On this basis, in December 2016, the General Office of the State Council further issued the “Several Opinions on Fully Opening the Older Adult Care Service Market and Improving the Quality of Older Adult Care Services,” emphasizing strong support for encouraging the design and development of smart products, health monitoring wearable devices, and health care mobile apps suitable for older adults. The implementation of this series of policies brought unprecedented development dividends to the smart older adult care industry, greatly stimulating market vitality and promoting the rapid development of the industry. By 2016, the market size of China's smart older adult care industry reached 1.31 trillion yuan, with a growth rate of 87.14% compared to the previous year, fully demonstrating the powerful driving role of policy guidance in the rapid development of the industry.

(2) Lack of momentum is the main reason for the gradual slowdown stage. In the early stages of the market, due to the presence of numerous gaps, many companies entered the market, collectively driving its rapid expansion. By 2018, the number of smart older adult care enterprises in China reached 887, representing a significant 46.13% increase compared to the previous year, and the market capacity reached a new peak. However, as the market gradually became saturated, these gaps were gradually filled by enterprises, and new growth points were difficult to emerge quickly. At the same time, market demand began to stabilize, with the growth rate of the older adult population being relatively limited. Consumers' demands and expectations for smart older adult care products also became more rational, no longer solely pursuing novelty and high-end features. This stabilization of market demand further intensified market competition, with an increasing number of enterprises, and competition across multiple dimensions, such as technology, quality, and service, became dominant. Against this backdrop, the growth rate of the smart older adult care market gradually leveled off, and the market entered a stage of development stagnation.

(3)Technological innovation is the key factor in the recovery stage. As the growth rate slowed down and market competition intensified, enterprises gradually realized that in order to stand out in fierce competition, they had to rely on technological innovation. By introducing advanced technologies such as artificial intelligence, big data, and the Internet of Things, smart older adult care enterprises could provide more precise and efficient older adult care services. For example, smart wearable devices could monitor the health status of older adults in real time, and remote medical services allowed older adults to enjoy professional medical care at home. By 2019, the number of smart older adult care products in China had reached 118, with a growth rate of 110.71% compared to the previous year, achieving exponential growth.

(4)The COVID-19 pandemic is a key factor in the continuous decline stage. During the pandemic, in order to effectively prevent the spread of the virus, older adult care institutions were forced to implement closed management measures, severely restricting the movement of people. While this greatly reduced the risk of the virus spreading within older adult care institutions and ensured the safety of older adults, it also directly led to a change in the supply-demand structure of the older adult care services market. As the main consumers of older adult care services, older adults found their daily lives and access to older adult care services greatly restricted under the closed management, no longer able to conveniently enjoy various older adult care services as they did before the pandemic. At the same time, the uncertainty and widespread fear brought about by the pandemic had a profound impact on the consumption psychology of older adults and their families. Faced with the unknown risks of the pandemic, they became more cautious and conservative when choosing older adult care services. Some families even temporarily abandoned older adult care services or drastically reduced their spending on older adult care services in order to lower the risk of infection. This clear reduction in consumption willingness further exacerbated the downward trend of the smart older adult care market.

The spatial differences in the coupling and coordinated development of China's digital economy and older adult care services are significant, forming a diverse spatial development pattern characterized by rapid growth in coastal areas, huge potential in inland areas, and relatively slow development in peripheral areas. At the same time, the geographic proximity effect is prominent, with neighboring areas interdependent and mutually promoting each other in the development process, forming a regional linkage development trend. The reasons for this phenomenon can be summarized as follows:

(1) Coastal areas, with their unique geographical advantages, actively engage in external exchanges and international trade. They have taken the lead in technological innovation and economic development, gradually accumulating a solid economic foundation, which provides favorable conditions for the integrated development of digital economy and older adult care services. As of 2022, the total GDP of coastal areas reached 63.57 trillion yuan, accounting for 52.53% of the national GDP. With the increasing degree of population aging, the demand for older adult care services is becoming more diversified and high-quality. The innovative capabilities and economic level in coastal areas can fully meet this market demand. As a result, coastal areas have achieved remarkable success in the coupling and coordinated development of the digital economy and older adult care services, becoming a national model.

(2) Although inland areas are relatively geographically isolated, their rich natural resources and human resources offer significant potential for the integrated development of digital economy and older adult care services. As of 2022, the population of inland areas reached 6.67 billion, accounting for 47.24% of the national population. At the same time, with the country's increasing focus on and support for inland development, infrastructure construction in inland areas is constantly improving, and technological innovation capabilities are gradually enhancing. As the degree of population aging gradually increases, the older adult care service market in inland areas will continue to expand, and the integration of digital economy and older adult care services will usher in new development opportunities.

(3) Peripheral areas, due to their remote geographical locations, relatively underdeveloped transportation and communication infrastructure, and weak economic foundation, as well as the evident monoculture of industrial structure, are severely limited in the integration and application of digital technology and economic development. Additionally, constrained by natural conditions such as climate, soil, and water resources, the population in peripheral areas is sparsely distributed. According to statistics, as of 2022, the average population density in China's peripheral areas was only 11.83 people per square kilometer, which is only 7.9% of the national average population density. This low population density further leads to relatively low levels of population aging in peripheral areas, resulting in insufficient market demand and further restricting the integrated development of the digital economy and older adult care services.

(4) Due to the similarity and complementarity in infrastructure such as transportation and communication between neighboring areas, it promotes the efficient flow and optimal allocation of production factors such as labor, capital, and technology between these areas. In this process, more developed areas can leverage their advantages to drive the progress of relatively underdeveloped areas. At the same time, underdeveloped areas can benefit from the radiation effect of more developed areas, seizing development opportunities to accelerate their own development. This positive interaction between regions helps achieve the circulation and sharing of resource factors, promoting the coordinated development of regions.

The coupling and coordinated development of China's digital economy and older adult care services are influenced by factors such as information communication, technological innovation, industry application, infrastructure, service capabilities, and organizational management. Among these, key drivers include the number of websites per 100 enterprises, the total value of technology contracts, the proportion of information technology service income in GDP, institutional building area, daily in-hospital visits, and the number of professional technical personnel. The reasons for this phenomenon can be summarized as follows:

(1) Number of websites per 100 enterprises measures the extent of digital transformation among local enterprises. As this indicator increases, more older adult care service enterprises establish online platforms to offer functions such as information consultation, service reservations, and health management. This not only enhances the convenience and accessibility of services but also enables older adult care services to achieve broader coverage and more efficient operations through online platforms, thus promoting the deep integration of older adult care services and the digital economy.

(2) Total value of technology contracts reflects the activity level of the local technology innovation market. An increase in the total value of technology contracts signifies the introduction and application of more advanced technologies and solutions. These technologies, such as smart wearable devices and remote medical monitoring, significantly improve the specialization of older adult care services and, through digital means, enable personalized and precise services, thus driving the deep integration of the digital economy and older adult care services.

(3) Proportion of information technology service income in GDP measures the contribution of the digital economy to the local economy. An increase in this proportion indicates the growing role of the digital economy in regional economic development. In the older adult care service sector, this means more resources are being invested in the digital transformation of enterprises, which strongly supports the innovation of older adult care service models and the improvement of service efficiency, providing solid support for the sector's transformation and upgrading.

(4) Institutional building area reflects the scale of older adult care service facilities. A larger institutional building area not only means that more older adults can be accommodated, providing them with more comprehensive services, but also provides the physical foundation necessary for the digital transformation of older adult care services. In such spaces, it becomes easier to install various smart devices and set up IoT facilities, thus improving the intelligence level of older adult care services, optimizing operational efficiency, and creating a more convenient and efficient environment for older adults.

(5) Daily in-hospital visits directly reflect the demand for older adult care services. An increase in daily in-hospital visits indicates sustained growth in the demand for older adult care services. To meet this demand, older adult care institutions need to continuously improve service efficiency and quality to better cater to the diverse needs of older adults. Digital transformation is the key path for institutions to achieve this goal. By introducing and applying advanced information technologies, institutions can optimize management processes, enhance operational efficiency, and provide more personalized and thoughtful services to older adults.

(6) Number of professional technical personnel reflects the level of specialization in the older adult care service industry. An increase in the number of professional technical personnel means a higher level of specialization in the sector. These professionals, with their deep knowledge and rich practical experience, can provide higher-quality and more specialized services to older adults. Their presence not only strengthens the service capabilities of older adult care institutions but also enhances the overall service standards and image of the industry, laying a solid foundation for the coordinated development of the digital economy and older adult care services.

### 5.2 Innovation

The innovation of this study lies in integrating the seemingly independent fields of the digital economy and older adult care services. By thoroughly analyzing their intrinsic connections and current development statuses, it aims to reveal the potential contribution of the digital economy to enhancing the quality and efficiency of older adult care services. Additionally, it explores how the demands of older adult care services can drive innovation and application of digital technologies. This interdisciplinary research not only enriches existing theories and promotes understanding of the integrated development of the digital economy and older adult care services but also provides new perspectives and data support for relevant policy formulation. This is crucial for building a more comprehensive and sustainable older adult care service system. Moreover, the study offers transferable insights for the application of the digital economy in other social service sectors, advancing society toward intelligent service transformation and striving to maximize dual economic and social benefits.

### 5.3 Limitations and future research directions

It is worth noting that this study may have certain limitations. In terms of temporal scale, data collection was only available up to 2022, with the latest year's data not yet obtained, which somewhat affects the timeliness and cutting-edge nature of the research findings. On the spatial scale, the analysis primarily focused on the provincial level and did not delve into more micro-levels such as city or county levels, thus limiting our ability to accurately capture and deeply explore the differences and characteristics of socioeconomic phenomena at finer spatial scales. Future research could refine these aspects by extending the analysis to the latest available year and expanding the scope to include more micro-levels such as cities and counties. This would help to more precisely reveal the differences and characteristics of socioeconomic phenomena among micro-level regions, thereby comprehensively understanding their developmental trends. Moreover, this approach could provide policymakers with more specific and targeted evidence, aiding in achieving precise governance and sustainable development of socioeconomic factors.

## 6 Conclusion and recommendations

### 6.1 Conclusions

This paper aims to explore the spatiotemporal evolution and driving factors of the coordinated development between China's digital economy and older adult care services. Official data from 2015 to 2022 were collected and analyzed using entropy method, coupling coordination degree model, and geographical detector model. The main conclusions are as follows: (1) The coupling and coordinated development of China's digital economy and older adult care services shows an upward trend, but with uneven growth, following an “M-shaped” fluctuation pattern of “rapid growth—gradual slowdown—bottoming out and recovery—continuous decline.” (2) There is notable spatial disparity in their development, with rapid progress in coastal areas, significant potential in inland regions, and slower development in peripheral areas. Geographical proximity also plays a key role, with neighboring regions interdependent and fostering mutual growth, creating a regional linkage trend. (3) Key factors influencing this development include the number of enterprise websites per 100 companies, total technology contract transactions, the share of IT service revenue in GDP, institutional building area, daily resident numbers in facilities, and the number of professional technical staff.

### 6.2 Policy recommendations

To achieve coordinated development between the digital economy and older adult care services, it is essential to analyze the inherent mechanisms of their integration and propose corresponding policy recommendations. Firstly, policy support is the fundamental guarantee for integrated development. During periods of rapid development, the government should introduce supportive policies such as research and development subsidies and tax reductions to encourage enterprises to increase their investment in research and development in the integration field, exploring new technologies and applications such as wearable devices and telemedicine services. Through technological innovation, the smart level of older adult care services can be enhanced, accelerating the integration process between the digital economy and older adult care services. In the phase of gradual slowdown, as the market gradually saturates and competition intensifies, the government should encourage enterprises to integrate and reorganize, increase industry concentration, create economies of scale, and guide companies to delve into niche markets, providing customized services.

Optimizing the industrial structure can improve the overall competitiveness of the industry and drive the sector toward high-quality development. In the phase of bottoming out and recovery, the government should encourage enterprises to explore new service models, such as the application of the sharing economy and platform economy in older adult care services, to expand market space and enhance the industry's innovation capabilities. By deepening innovation and development, new vitality can be injected into the sector, promoting new growth. In the phase of continuous decline, the government should closely monitor market dynamics, analyze the reasons for the industry's decline, and adjust relevant policies in a timely manner according to the actual situation. For example, providing transformation assistance to guide the industry to expand into emerging fields, strengthening regulation to ensure market order, and increasing publicity efforts to enhance social awareness and acceptance. By adjusting policies in a timely manner, the government can help the industry face challenges and achieve sustainable development.

Secondly, market demand is the intrinsic driving force for integrated development. Coastal areas, with their unique geographical advantages, have established an open external environment and nurtured a relatively mature market system. These foundational conditions greatly stimulate the urgent demand for improving the quality of older adult care services. Driven by this strong market demand, coastal areas should focus on cultivating and introducing leading enterprises in the digital economy, constantly updating ideas and technologies, and applying them to the older adult care sector, further enhancing the intelligence and specialization of older adult care services. In contrast, inland areas, while rich in natural and human resources, have a slower pace of economic development. As the aging process accelerates, the demand for older adult care services is becoming increasingly urgent, which endows inland areas with tremendous development potential in the older adult care market.

Driven by such market demand, inland areas should explore and fully utilize their own potential, relying on abundant natural resources, and explore development paths for the integration of digital economy and older adult care services with distinctive local characteristics, such as emerging models like smart tourism and wellness. At the same time, inland areas should actively strengthen exchanges and cooperation with coastal areas, learn from their successful experiences and development models, promote inter-regional collaboration, and jointly push forward the coordinated development of the digital economy and older adult care services to a new level. However, marginal areas, due to their lower population density and relatively underdeveloped infrastructure, have relatively limited market demand for older adult care services, and older adult care resources are also scarce. In this regard, marginal areas should base their efforts on local realities, ensuring that the core needs of older adults, such as basic daily care and medical services, are prioritized.

To this end, marginal areas can establish community older adult care service centers, promote home-based older adult care services, and provide door-to-door services to offer convenient and efficient older adult care. These initiatives not only help meet the basic needs of older adults but also lay a solid foundation for the sustainable development of the older adult care services market in marginal areas.

Thirdly, technological innovation-driven development is the key engine for integrated progress. As an important platform for enterprises to showcase their services and interact with customers, the ownership of corporate websites directly reflects the degree of external openness and the level of digital construction of the enterprise. Therefore, we should further strengthen the application of technological innovation in the development of corporate websites, optimizing website functionality and enhancing user experience. This will enable older adult care institutions to comprehensively display service information online, facilitate convenient and efficient appointment services, and enhance interaction and communication with older adults and their families, thereby building closer service relationships.

The value of technology contracts is an important indicator of the market application of technological innovation achievements. Thus, we should vigorously support technological innovation cooperation between enterprises and leverage technology contracts as an effective vehicle to deeply integrate cutting-edge technologies such as smart wearable devices, telemedicine, and health monitoring into the older adult care sector. This will accelerate the transformation of research and development achievements into actual services to meet the increasingly diverse older adult care needs. The proportion of revenue from information technology services is a key indicator for measuring the effectiveness of digital transformation in enterprises. Therefore, we should place special emphasis on the core driving role of technological innovation. By widely applying advanced technologies such as cloud computing, big data, and the Internet of Things, we can achieve automation and intelligent transformation of service processes, significantly improving service efficiency and quality, and providing older adults with higher-quality and more efficient older adult care services. The building area of older adult care institutions is directly related to the quality and efficiency of service provision. Therefore, we should intensify innovation efforts, actively introduce advanced architectural design concepts and intelligent management systems, maximize the utilization of space, and optimize resource allocation, thereby enhancing the comfort of living and the quality of services in older adult care institutions. The daily number of residents in the institution is an important indicator for measuring the service capacity of the institution. Therefore, we should fully leverage the advantages of technological innovation. By introducing intelligent appointment systems, online service platforms, and other technological tools, we can achieve an upgrade of services to be more convenient and efficient, further improving the service experience and satisfaction of older adults.

Finally, talent cultivation is an important support for integrated development. Talents who possess both digital skills and professional knowledge in older adult care services are indispensable key forces in driving this integration process. On one hand, the government should increase investment in talent cultivation within the older adult care service sector by establishing special funds and providing training subsidies as incentive measures. This would encourage and support universities, vocational colleges, and training institutions to offer courses closely related to older adult care services, focusing on cultivating professionals who are skilled in digital technologies while adhering to older adult care service principles. On the other hand, older adult care institutions themselves should place great emphasis on building their talent teams.

Not only should they improve the digital literacy and older adult care service skills of existing employees through internal training, but they should also regularly organize digital technology training courses, such as big data analysis, cloud computing applications, and smart device operation, to ensure employees stay up to date with the latest technological advancements and apply these digital technologies in older adult care practice. Additionally, they should actively recruit professionals with digital skills and older adult care experience through external recruitment and establish close partnerships with universities to bring in outstanding interns and graduates, continuously injecting new vitality into the institutions. This will drive the deep integration of the digital economy with older adult care services and promote high-quality development.

## Data Availability

The original contributions presented in the study are included in the article/supplementary material, further inquiries can be directed to the corresponding author.
